# Supplementary material for: Angiosperm speciation cools down in the tropics
Source: Ecol Lett. 2020 Feb 11;23(4):692–700. doi: 10.1111/ele.13476 (PMC7078993; doi:10.1111/ele.13476)
Supplement: Supplementary file 1 [file ELE-23-692-s001.docx]

| dataset name | λ_temperate_ | λ_tropical_ | p-value | dataset size |
| --- | --- | --- | --- | --- |
| more.GBIF.data | 0.702 | 0.641 | 0.050 | 53,344 |
| no.widespread | 0.760 | 0.668 | 0.064 | 46,426 |
| no.high.latitude | 0.719 | 0.652 | 0.044 | 60,034 |

**Table S1.** Temperate species have mean higher speciation rates (λ, estimated with STRAPP) than tropical species across different datasets: “more.GBIF.data” excludes species with less than five GBIF data points; “no.widespread” excludes species occurring both in and outside the tropics; “no.high.latitude” excludes species from poorly sampled latitudinal bands with absolute median latitude ≥50°. Units for λ and dataset size are lineages/myr and species, respectively.

**Supplementary Note 1**

Smaller seeded species have been shown to speciate faster than large seeded species (Igea *et al.* 2017). We assessed whether seed size differed between temperate and tropical lineages as it has previously been shown to do so (Moles *et al.* 2007), and so latitudinal variation in seed size could explain the differences in speciation between tropical and temperate species. We obtained seed size measurements for 13,178 species in our full dataset from a previous study (Igea *et al.* 2017) and we found that seed size was larger in tropical species (mean seed size_temperate_ = 0.0018 g; mean seed size_tropical_ = 0.0125 g; t = -28.239; df = 4300.7; p-value < 0.001). This difference remained when phylogeny was considered (phylANOVA: t = 33.933; p value = 0.001; significance assessed with 1,000 random simulations using phytools (Revell 2012))

**Supplementary Note 2**

BAMM is a model-based approach to estimate tip speciation rates. To compare the BAMM results with a non-model based approach (i.e., that only relies on branch lengths and splitting events), we calculated the Diversification Rate metric (DR; Jetz *et al.* 2012) for the species in our full dataset (n = 60,990). This measure of recent speciation rate incorporates the number of nodes and the internode distances separating a species from the root and gives greater weight to branches closer to the present (Title & Rabosky 2019). We then compared the DR values of tropical and temperate species and assessed the variation of DR across latitudinal bands. Mirroring the BAMM results, we found that tropical species had smaller values of DR and that latitudinal bands closer to the poles had higher values of DR (Fig. S9).

**
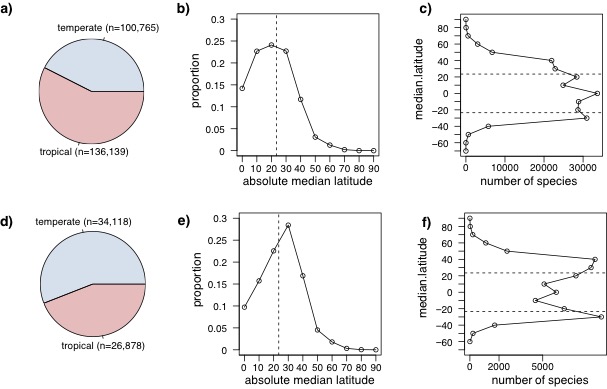
Figure S1.** The full dataset had a disproportionate number of temperate species. **a)** Proportion of tropical and temperate species in the GBIF dataset (n = 236,894); **b)** proportion of species in each latitudinal band in the GBIF dataset; **c)** number of species in each latitudinal band in the GBIF dataset; **d)** proportion of tropical and temperate species in the full dataset (n = 60,990); **e)** proportion of species in each latitudinal band in the full dataset; **f)** number of species in each latitudinal band in the full dataset; in **b), c),** **e)** and **f)**, the dotted vertical line denotes the 23.5° threshold between tropical and temperate zones.


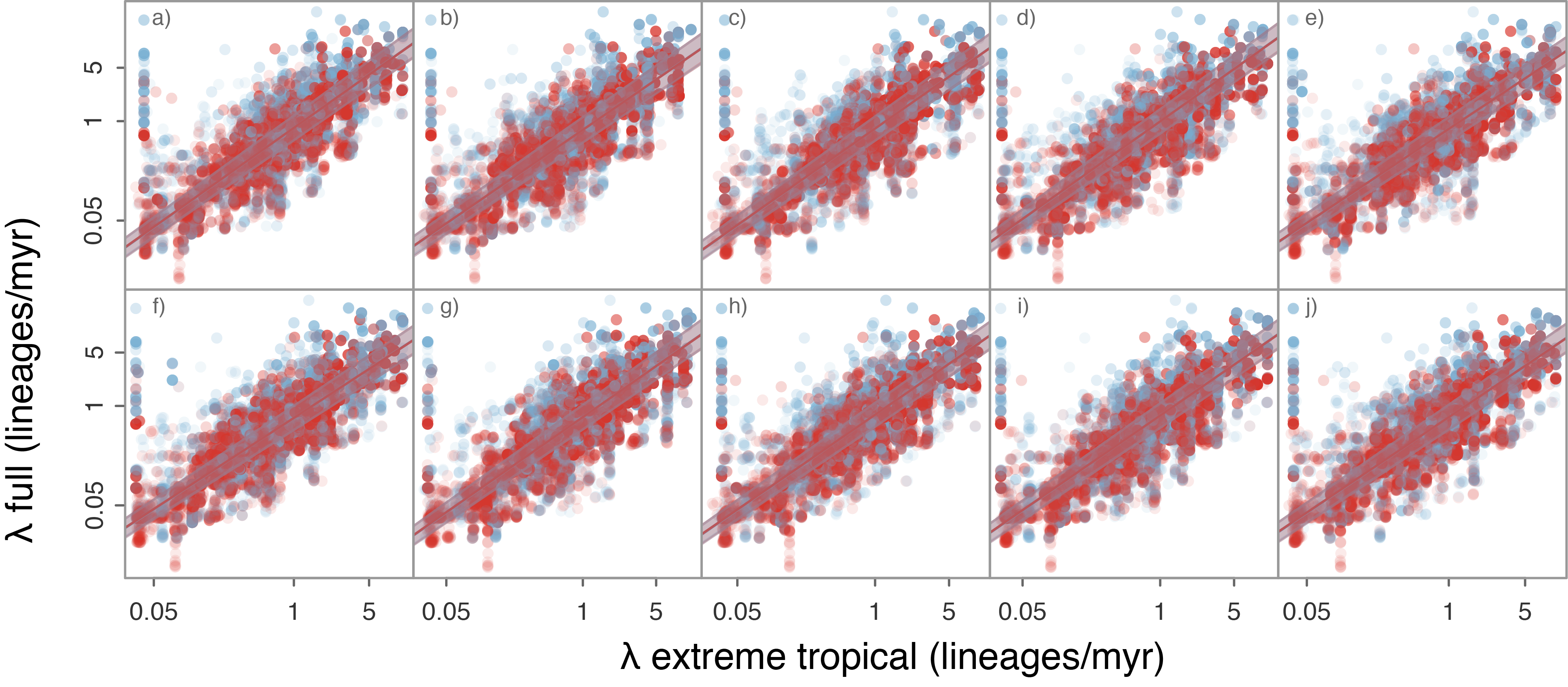


**Figure 2. BAMM λ estimates were positively correlated in the full and 10 extreme tropical datasets (a to j) with no effect of tropicality on this correlation**. Solid lines are phylogenetic linear regressions predicting λ in the full tree (n = 60,990 species) with λ in the extreme tropical tree (n = 30,000 species) in temperate (blue) and tropical (red) species. Shaded areas indicate the 95% confidence intervals.

**
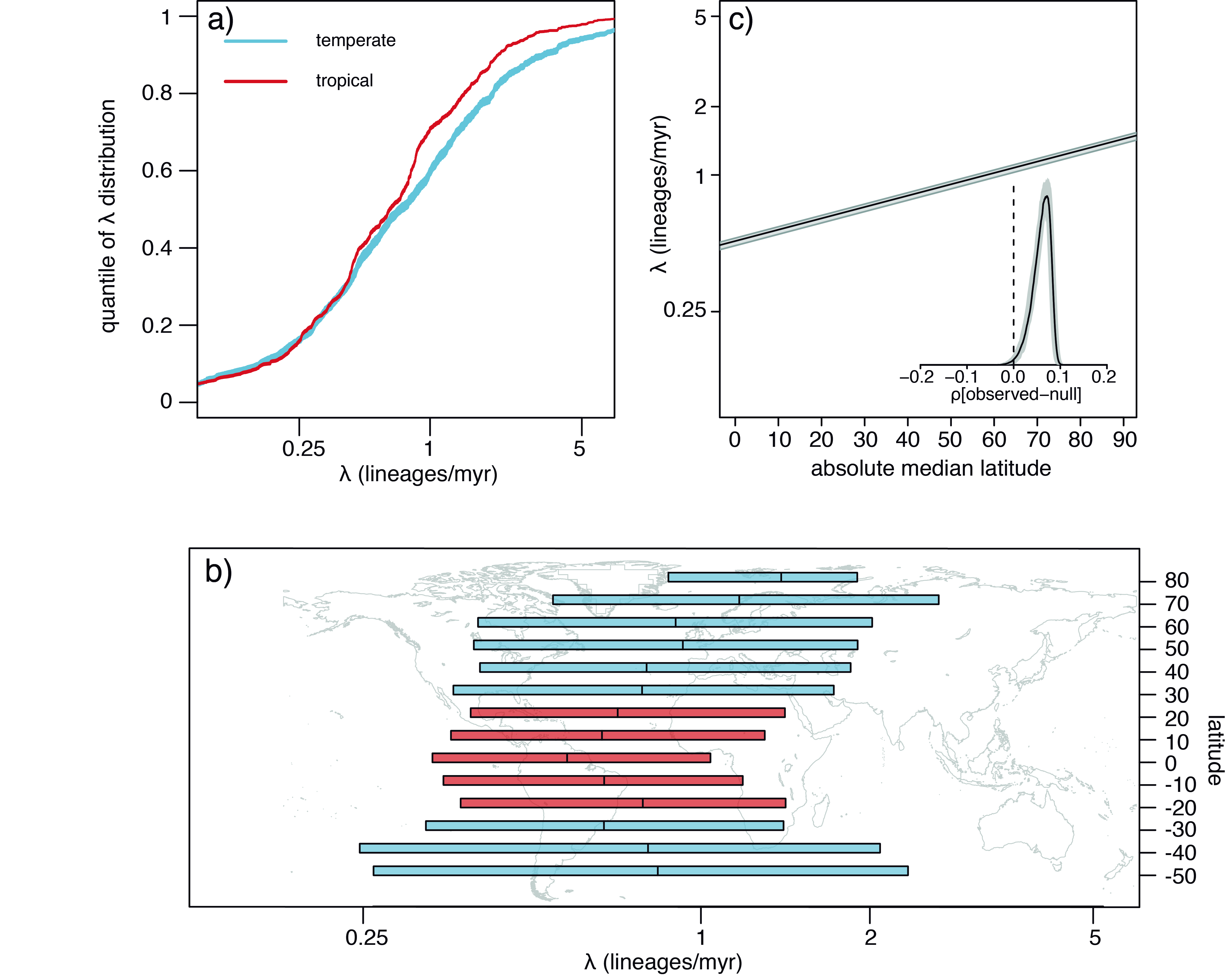
**

**Figure S3. Temperate species have higher speciation rates (λ) than tropical species for 100 subsampled “extreme tropical” datasets with 29.1% temperate and 70.9% tropical species**. **a)** Rank ordered distribution of λ inferred with BAMM for tropical (red) and temperate (blue) species. **b)** λ grouped by latitudinal band of each species. Boxes and lines are presented as in Fig. S4. **c)** Spearman’s ρ correlation of species absolute median latitude and λ as estimated with STRAPP. . Solid black line indicates median correlation ± 95% confidence interval across the posterior distribution. The inset shows the median (black line) ± 95% confidence interval for the difference between the empirical and null correlations estimated with 1000 permutations of evolutionary rates across the phylogeny in each of 100 subsampled datasets.


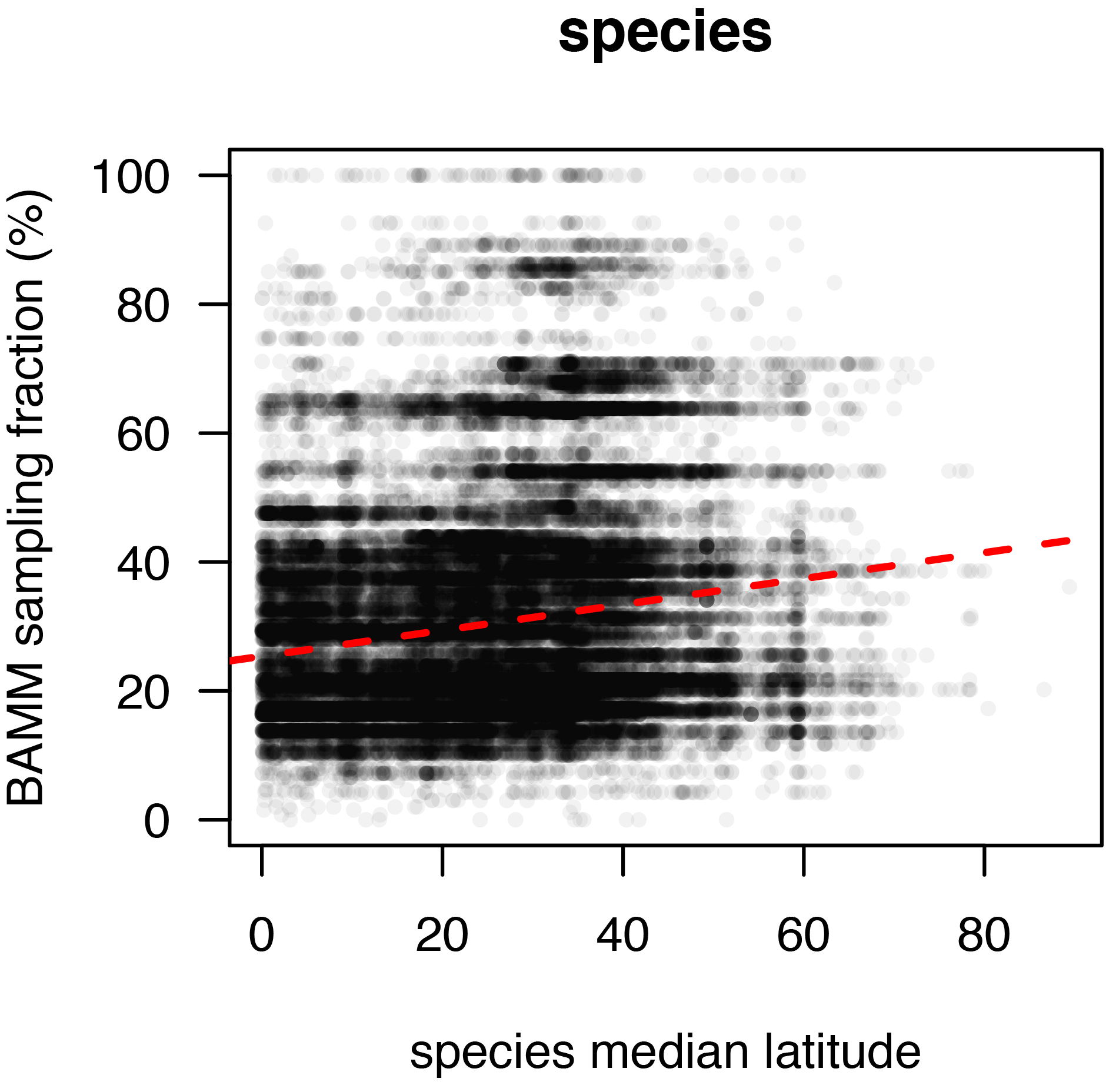


**Figure S4.** Relationship between the species absolute median latitude and the family-level sampling fraction used in the BAMM analyses. The red line is the slope of the linear regression (slope = 0.201, p-value < 0.0001).


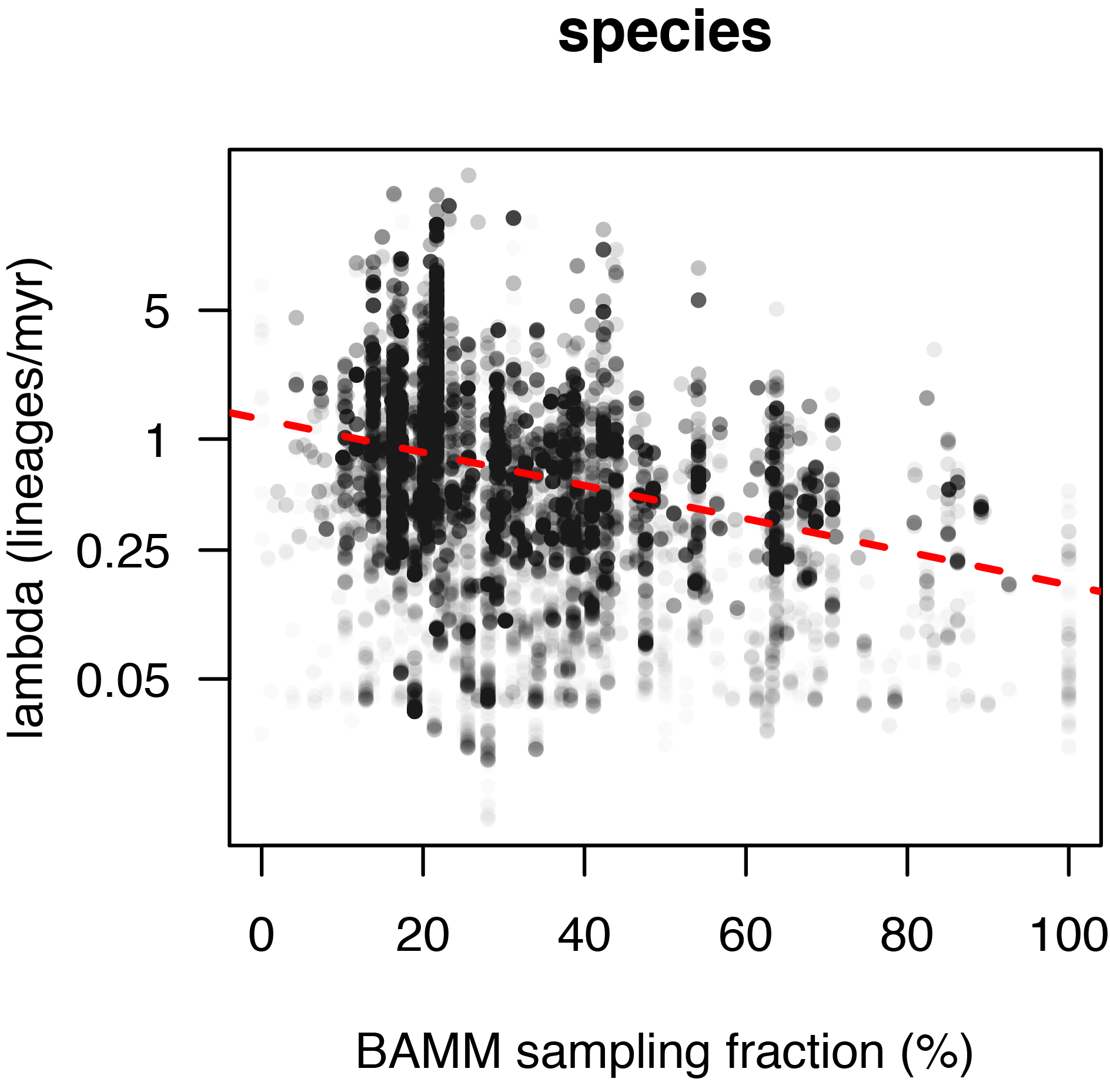


**Figure S5.** More densely sampled clades have smaller estimates of speciation rate (λ). The dotted line is the slope of the linear regression of the log(λ) and the BAMM sampling fraction (slope = -0.027 , p-value < 0.0001).

**
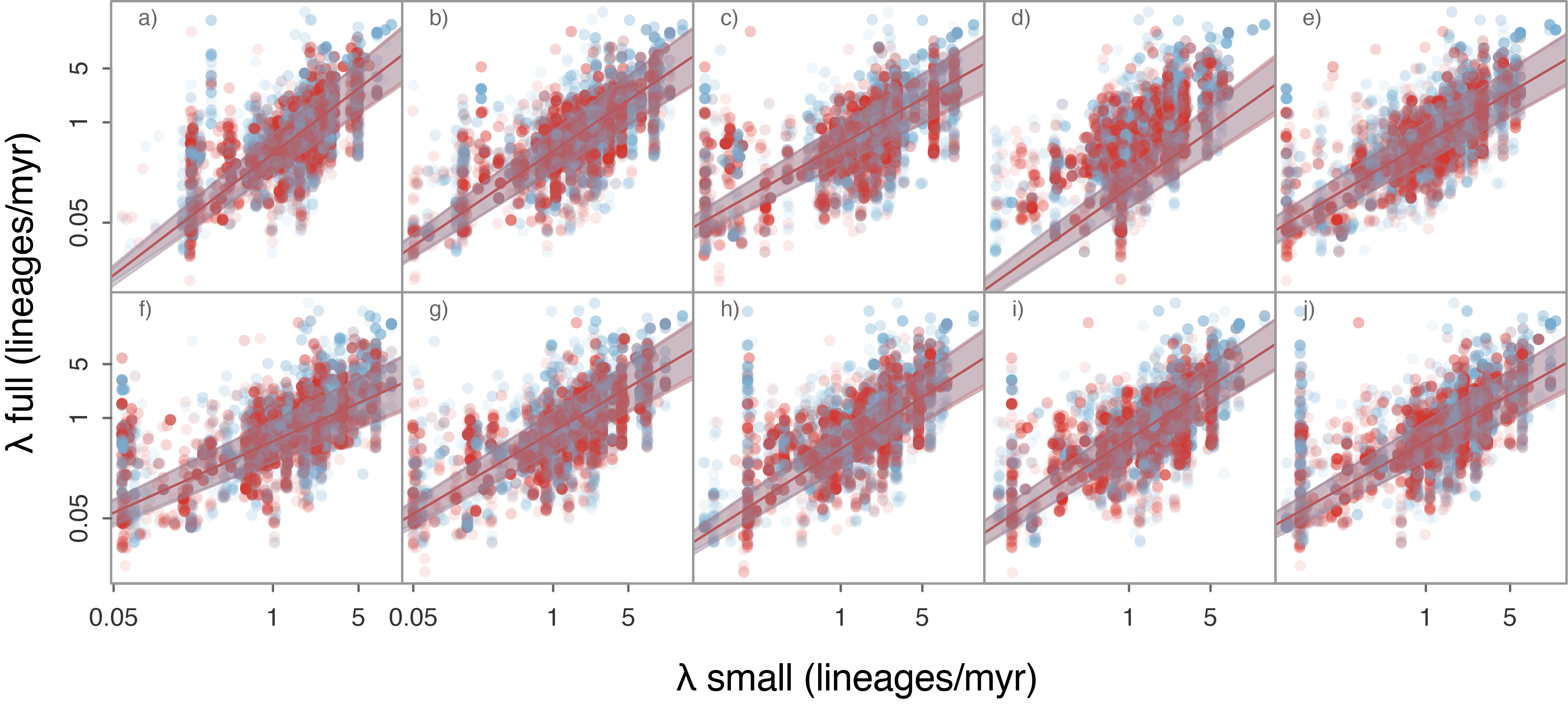
Figure S6.** BAMM λ estimates are positively correlated in the full and 10 small datasets and tropicality has no effect on this correlation. **a) - j)** Solid lines are phylogenetic linear regressions predicting λ in the full tree (n = 60,990 species) with λ in the small tree (n = 10,739 species) in temperate (shown in blue) and tropical (shown in red) species. Shaded areas indicate the 95% confidence intervals.

**Figure S7.** Tropical species have smaller speciation rates (λ) than temperate species with the Zanne phylogeny (n = 28,057 species). a) Rank ordered distribution and boxplot (inset) of λ inferred with BAMM for tropical (red) and temperate (blue) species; b) λ grouped by latitudinal band of each species; and c) Spearman’s ρ correlation between species absolute median latitude and λ as estimated with STRAPP. Lines, boxes and symbols as in Fig. 1.

**Figure S8.** Temperate clades have higher speciation rates in the clade-based analysis. Correlation of a) the proportion of tropical and b) temperate species in each clade with the corresponding speciation rate (λ) estimated with RPANDA. The correlation coefficient is the phylogenetic generalised least squares (PGLS) slope. The size of the points is scaled to the number of clades in each time interval and their colours show the statistical significance of the slope.

**
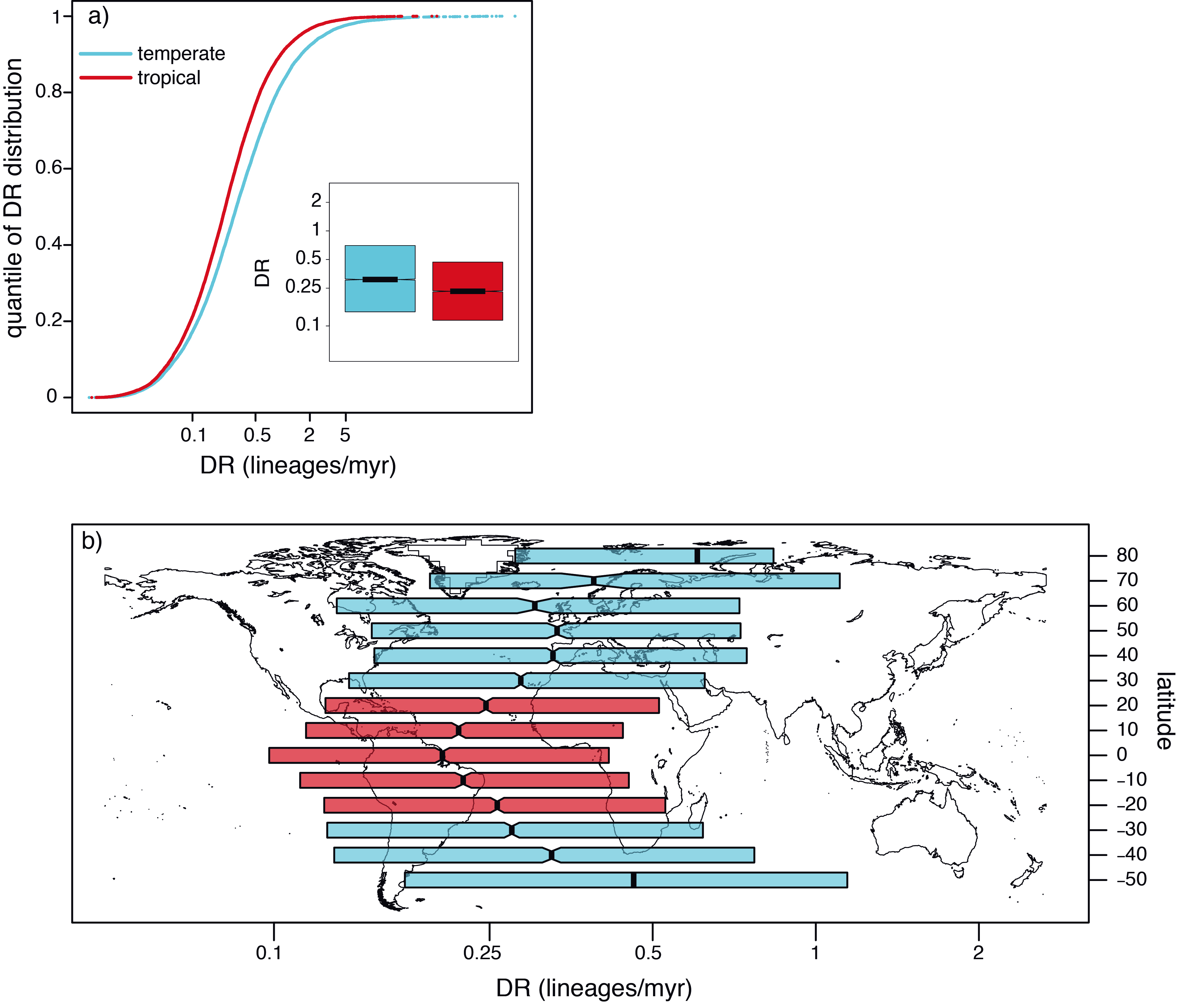
**

**Figure S9. Tropical species have smaller DR values than temperate species. a)** Rank-ordered distribution and boxplot (inset) of DR for tropical (red) and temperate (blue) species. **b)** DR grouped by latitudinal band of each species. Notches in boxplots indicate 95% confidence intervals around median, denoted by thick vertical lines and boxes span the interquartile ranges.
